# Alirocumab *versus* Evolocumab on Cardiovascular Outcomes: A Systematic Review and Meta-analysis

**DOI:** 10.2174/011573403X357542250526072430

**Published:** 2025-06-10

**Authors:** André Saad Cleto, João Matheus Schirlo, Janete Machozeki, Camila Marinelli Martins

**Affiliations:** 1 Department of Medicine, State University of Ponta Grossa, Ponta Grossa, Brazil

**Keywords:** Alirocumab, evolocumab, cardiovascular outcomes, PCSK9 inhibitors, hypercholesterolemia, LDL-C receptors

## Abstract

**Introduction:**

The PCSK9 enzyme is present mainly in the liver and is responsible for the degradation of LDL-C receptors. Currently, there are some drugs that inhibit this enzyme, such as alirocumab and evolocumab. Consequently, these drugs reduce serum LDL-C levels. Therefore, a systematic review and a meta-analysis were carried out in order to compare alirocumab against evolocumab in reducing cardiovascular outcomes.

**Methods:**

This systematic review was carried out in accordance with PRISMA and was registered in PROSPERO (CRD42024573217). The following databases were searched on July, 9, 2024: PubMed, Web of Science and Scopus. Randomized clinical trials with a control group were included and meta-analyses were performed to assess relative risk (RR). The random effects model was used in heterogeneous samples. The articles were distributed into 2 subgroups: use of alirocumab and evolocumab.

**Results:**

Initially, 2,213 articles were found, of which 6 were included. In total, 62,119 patients participated. The RR values ​​were significant for alirocumab in the following outcomes: myocardial infarction (MI) 0.85 (95% CI 0.77-0.93), stroke 0.75 (95% CI 0.60-0.94) and hospitalization for unstable angina 0.58 (95% CI 0.39-0.86), while for evolocumab they were significant for MI 0.75 (95% CI 0.68-0.83) and coronary revascularization 0.81 (95 CI % 0.75-0.88). There was a statistically significant difference between the drugs for hospitalization for unstable angina (*p*=0.02).

**Discussion:**

This study highlights the benefits of PCSK9 inhibitors, especially alirocumab, in reducing major cardiovascular events. Alirocumab significantly lowered hospitalizations for unstable angina, with a 42% reduction, and showed favorable outcomes in reducing myocardial infarction, coronary revascularization, and stroke. These reductions are clinically meaningful, as they lower morbidity, improve patient quality of life, and reduce healthcare costs. Both alirocumab and evolocumab are effective and safe, offering important therapeutic options for high-risk cardiovascular patients.

**Conclusion:**

The use of alirocumab is preferable if the focus is to avoid hospitalizations for unstable angina or stroke, while evolocumab may be an option if one wants to avoid coronary revascularization. Both drugs are effective in reducing cardiovascular outcomes, but alirocumab was superior to evolocumab.

## 1 INTRODUCTION

The proprotein convertase enzyme subtilisin/kexin type 9 (PCSK9) is responsible for degrading LDL-C receptors present mainly in hepatocytes. This action has the main consequence of reducing the removal of LDL-C from the bloodstream. Currently, PCSK9 inhibitors are a class of drugs that act by inactivating this enzyme [1]. Such inactivation can be carried out by human monoclonal antibodies, something that is carried out by alirocumab and evolocumab [2, 3], as well as by silencing the translation of the mRNA that forms the enzyme, an action which is carried out by inclisiran [4]. Therefore, the action of these drugs causes important reductions in serum LDL-C levels [2-4]. In a real-world setting, PCSK9 inhibitors were able to lead to a 70% reduction in LDL-c levels when used as an intensive and early lipid-lowering therapy in patients with acute coronary syndrome [5]. In this sense, the 2023 ESC Guidelines for the management of acute coronary syndromes suggest the use of PCKS9 inhibitors for patients who do not achieve LDL-C goal despite maximally tolerated statin therapy and ezetimibe after 4-6 weeks [6].

LDL-C is an important risk factor for the establishment of cardiovascular diseases [7], which are considered the biggest cause of death worldwide [8]. PCSK9 inhibitors were able to reduce some cardiovascular outcomes [1-3, 9-11]. Additionally, evolocumab has been associated with a reduction in atherosclerotic plaques in coronary arteries [12]. These results are extremely important for clinical practice since the reduction in LDL-C, associated with a reduction in cardiovascular outcomes, is extremely relevant in patients at high cardiovascular risk [12]. There are a greater number of randomized clinical trials that used alirocumab or evolocumab instead of inclisiran. Despite the clinical relevance of Inclisiran, no studies have yet been published evaluating its impact on cardiovascular outcomes. Dispite of these suggestive data in clinical studies, more evidence is still needed with meta-analysis studies to consolidate the effect of evolucumab and alirocumab on cardiovascular outcomes. Therefore, the objective of this systematic review with meta-analysis is to evaluate whether alirocumab is superior to evolocumab in reducing cardiovascular outcomes.

## 2 MATERIALS AND METHODS

### 2.1 Eligibility Criteria

This systematic review was registered on the PROSPERO platform (CRD42024573217) and was carried out in accordance with the Preferred Reporting Items for Systematic Review and Meta-Analysis Protocols Statement (PRISMA) [13]. Only randomized clinical trials with a control group were included, in which participants of any age had high cardiovascular risk or hypercholesterolemia (LDL-C>100 mg/dl). The treatment used was alirocumab or evolocumab, subcutaneously, regardless of the dosage or frequency of administration of the drug. Studies presented data on the number of events for at least 1 of the following cardiovascular outcomes: acute myocardial infarction (AMI), stroke, hospitalization for heart failure (HF) or unstable angina, coronary revascularization and death from cardiovascular causes. Clinical trials that provided data on deaths from any cause were also included. There were no restrictions related to the date of publication of the article nor the original language in which these works were published. Studies that only presented the study design or that were about cost-effectiveness were excluded, as were any other type of study that was not a randomized clinical trial with a control group. The studies were grouped according to the drug used.

### 2.2 Information Sources and Search Strategy

The search for articles was carried out on 07/09/2024 in the PubMed, Scopus and Web of Science databases. The descriptors used were: (“Alirocumab” OR “Evolocumab”) AND cardiovascular. An additional search in other databases was carried out to find other clinical trials that used Evolucumab or Alirocumab and reported cardiovascular outcomes with no other results.

### 2.3 Selection and Data Collection Process

Initially, the articles were exported from the databases to the Mendeley platform. Subsequently, successive stages of removing duplicates, reading titles, reading abstracts and complete reading of articles were carried out by 2 independent evaluators. In case of disagreement regarding the inclusion of an article after complete reading, a third evaluator would be called to make the final decision. Inclusion and exclusion criteria were applied at each stage and no automated tools were used.

### 2.4 Data Extraction

The following data were extracted: corresponding author, country where the study was carried out, year of publication, average age, sex, body mass index (BMI), glycated hemoglobin (HbA1c), frequency of administration of evolocumab or alirocumab, average follow-up time and cardiovascular outcomes (myocardial infarction, stroke, hospitalization for heart failure or unstable angina, coronary revascularization, death from cardiovascular causes and death from any cause). Such data were extracted and stored in Google Spreadsheets by 2 independent evaluators.

### 2.5 Study Risk of Bias Assessment

The risk of bias was assessed using the RoB 2 tool [14]. This tool divides bias analysis into 5 aspects: randomization process, intervention, description of results, measurement of results and selection of reported results. An evaluator was responsible for answering several questions about the domains in this tool, which generates one of the following 3 conclusions: low risk of bias, some concerns and high risk of bias.

### 2.6 Effect Measures and Synthesis Methods

A descriptive analysis was carried out with the patients' age, sex and frequency of drug administration. Subsequently, meta-analysis models were carried out to assess relative risk with all previously described cardiovascular outcomes. These outcomes were grouped according to the medication used. Such meta-analysis models use the inverse variation method for binary outcomes.

The results of each study were combined into a single measurement and 95% CI. Single-measure estimates represented the weighted average of the frequency of cardiovascular outcomes from these studies and the calculation of the measurement confidence interval considering intra- and inter-study variability. Therefore, the representation of measurements obtained was presented through a “forest plot”. I^2^ tests were performed to assess heterogeneity and were considered significant at *p*<0.05. If the sample was heterogeneous, the random effects model was considered, whereas, in homogeneous samples, the result of the common effect model was taken into account. This was done because the random effects model mitigates differences between heterogeneous studies. Therefore, the use of such a model in this situation brings robustness to the results.

Furthermore, tests to assess differences between subgroups were performed and considered significant if *p*≤0.05. “Funnel plots” were used to evaluate publication bias. The meta-analyses used the “meta” package in the R 4.0.4 environment [15].

## 3 RESULTS

### 3.1 General Characteristics of the Included Studies

**Fig. (1):**
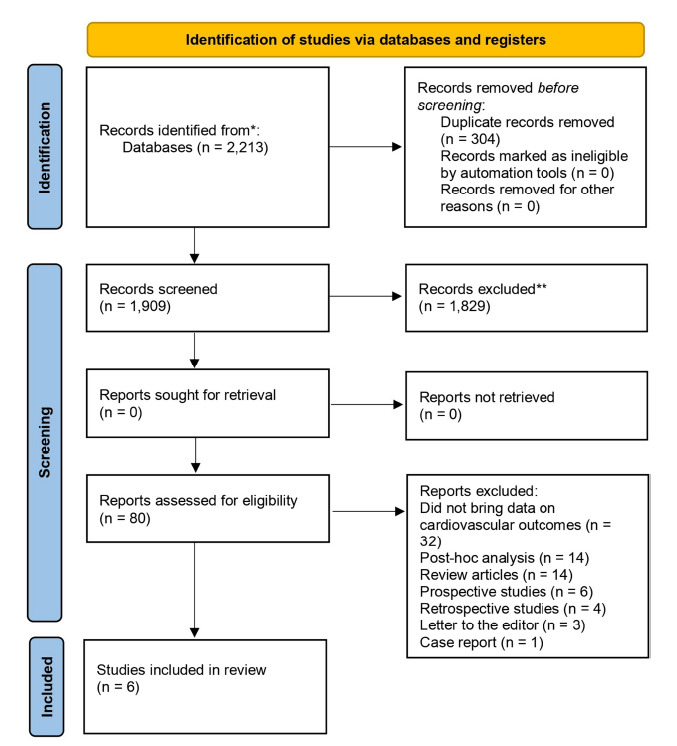
PRISMA flowchart.

Five hundred thirty articles were found in Web of Science, 1118 in Scopus and 565 in PubMed, totaling 2213 articles. Subsequently, 304 were excluded for being duplicates, 1829 after reading titles and abstracts and 74 were excluded after reading in full for the following reasons: they did not contain data on cardiovascular outcomes (32), post-hoc analysis (14), review articles (14), prospective studies (6), retrospective studies (4), letter to the editor (3) and case report (1). Therefore, 6 articles were included in this systematic review, 3 of which used alirocumab and 3 evolocumab (Fig. **1**).

All studies were multicenter and published in English. The publication period ranged from 2015 [9] to 2023 [10], while the average age ranged from 57.8 years [9] to 62.5 years [3]. The total number of patients was 62,119, ranging from 2,190 [10] to 27,564 [3]. The alirocumab doses used were 75 mg and 150 mg administered every 2 weeks, while the evolocumab doses used were 140 mg every 2 weeks or 420 mg once a month. The average follow-up time for patients was 2.41 years, ranging from 0.925 years [9] to 5 years [11]. The participants' average BMI was 28.6 kg/m^2^ and only one study provided values ​​for the average HbA1c, which was 5.9%^8^ (Table **1**).

### 3.2 Risk of Bias Assessment

**Fig. (2):**
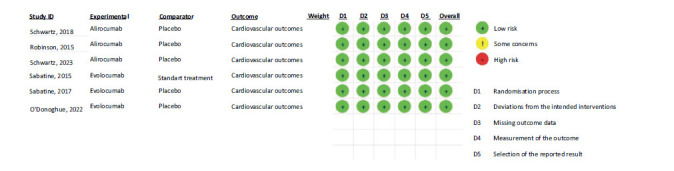
Risk of bias assessment.

The risk of bias was assessed using the RoB 2 tool [14]. All studies presented a low risk of bias in all domains analyzed by the tool. Therefore, all articles were classified as low risk of bias. The funnels did not reveal publication bias in the meta-analyses (Fig. **2**).

### 3.3 Meta-analysis

Individual meta-analyses of both drugs presented *p*-values ​​in heterogeneity tests greater than 0.05. Therefore, the common effects models were analyzed. Furthermore, the heterogeneity test values ​​of the two combined samples were statistically significant for death from any cause (*p*=0.02) and coronary revascularization (*p*=0.01). Therefore, in these meta-analyses, tests of differences between subgroups of the random effect were taken into account, whereas, for the other cardiovascular outcomes, the common effects model for the difference between subgroups was considered.

The results of the meta-analyses with alirocumab will be presented first, followed by the results with evolocumab.

Regarding AMI, the relative risks (RR) found were 0.85 (95% CI 0.77-0.93) and 0.75 (95% CI 0.68-0.83) (*p*=0.09), while the relative risk for stroke was 0.75 (95% CI 0.60-0.94) and 0.87 (95% CI 0.75-1.01) (*p*=0.28). There was no statistically significant difference between the drugs for these outcomes. The relative risk for coronary revascularization was 0.91 (95% CI 0.83-1.00) and 0.81 (95% CI 0.75-0.88). No significant difference was found between alirocumab and evolocumab for this outcome.

**Fig. (3):**
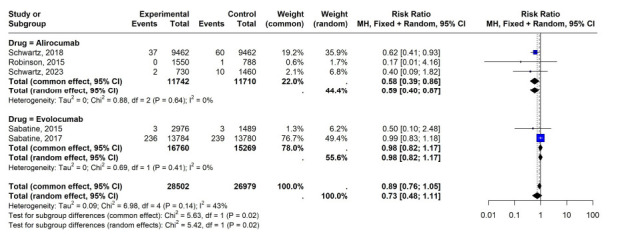
Hospitalizations for unstable angina.

Hospitalization for HF had results of 1.00 (95% CI 0.81-1.22) and 0.90 (95% CI 0.72-1.11) (*p*=0.49), while meta-analyses on deaths from cardiovascular causes and from any cause obtained the following results: 0.95 (95% CI 0.82-1.10) and 0.87 (95% CI 0.74-1.02) (*p*=0.40), 0.82 (95% CI 0.74-1.02) and 1.02 (95% CI 0.93-1.12) (*p*=0.08), respectively. There was no statistically significant difference between the drugs in these outcomes. Hospitalization for unstable angina had a relative risk of 0.58 (95% CI 0.39-0.86) and 0.98 (95% CI 0.82-1.17) (*p*=0.02). There was a statistically significant difference between drugs in reducing hospitalizations for unstable angina (Fig. **3**).

## 4 DISCUSSION

Other systematic reviews on the use of PCSK9 inhibitors have been previously published [16, 17]. However, this is the first systematic review with meta-analysis to directly compare the effectiveness of alirocumab *versus* evolocumab in reducing cardiovascular outcomes.

Alirocumab was statistically superior to evolocumab in reducing hospitalizations for unstable angina. This is relevant not only because of a *p*-value <0.05 but also because alirocumab achieved a clinically significant reduction of 42%. We did not find pharmacokinetic differences that justify the superiority of alirocumab. However, studies with evolocumab included more smokers, and people with diabetes and hypertension than clinical trials with alirocumab. This may explain the differences in cardiovascular outcomes found since these are variables little influenced by lipid metabolism, which is the site of action of PCSK9 inhibitors.

The action of alirocumab and evolocumab goes beyond cardiovascular outcomes. In a systematic review with a recent meta-analysis, alirocumab was superior to inclisiran in improving the lipid profile, especially in reducing LDL-C -51.74% *vs.* -41.34%, triglycerides -12.99% *vs.* -9.99% and total cholesterol -32.84% *vs.* -19.60% at 24 weeks [18]. Furthermore, a study with rats found a reduction in ischemic injury and neurological deficits after a stroke, something that raises the hypothesis that this class of drugs is effective in the acute phase of stroke [19, 20]. Another study investigated in a real-world setting the use of Evolucumab in the acute phase of ischemic stroke patients with a very high risk of atherosclerotic cardiovascular disease, obtaining a reduction in the recurrence of cerebrovascular events [21].

The atherosclerotic process is directly related to plasma lipoproteins, especially LDL-C. The PCSK9 enzyme participates in the LDL-C metabolism process and is produced mainly by the kidneys, liver and intestine. However, PCSK9 is produced by endothelial and smooth muscle cells of blood vessels and, to a lesser extent, by macrophages. This enzyme acts in a paracrine manner on macrophages, reducing the expression of LDL-C receptors on their surface and, consequently, reducing the formation of foam cells [22], which play a fundamental role in the formation of atherosclerotic plaque [23]. Therefore, it is clear that this is one of the factors responsible for the effectiveness of PCSK9 inhibitors in reducing cardiovascular outcomes since the formation of atherosclerotic plaque is fundamental for the development of cardiovascular diseases.

Factors other than the formation of atherosclerotic plaque are necessary for the development of cardiovascular diseases, one of these components being platelet aggregation. PCSK9 acts by increasing platelet aggregation. This action can be explained by the activation of Cyclooxygenase-1 (COX-1), which culminates in the formation of thromboxane A2. Some clinical trials that used alirocumab or evolocumab showed a reduction in platelet activation and aggregation [22]. Furthermore, it is known that the accumulation of platelets is related to the initial process of atherosclerosis and blood clotting after the rupture of the atherosclerotic plaque [23]. Therefore, it is possible to state that PCSK9 inhibitors have several pathways of action in reducing cardiovascular events.

Some recent studies have found a relationship between an increase in PCSK9 synthesis and the development of non-alcoholic fatty liver disease (NAFLD). With this in mind, Shafiq *et al*. conducted a retrospective study evaluating the efficacy of PCSK9 inhibitors in resolving NAFLD. In this study, 72.3% of patients had complete resolution of the condition, with an average time of using the drugs of 17.6 months [24]. However, it is necessary to carry out randomized clinical trials on the topic in order to carry out a study with a higher level of scientific evidence.

Alirocumab can be used in the treatment of familial hypercholesterolemia [25-31]. This drug was tested mainly in adults with the heterogeneous variant of the disease; however, randomized clinical trials were carried out with children, in which the efficacy and safety of the medication were verified [1]. Evolocumab was also tested in patients with familial hypercholesterolemia. Like alirocumab, it was also effective and safe in the treatment of this disease, both in adults and children [32-38]. There was no difference between the two PCSK9 inhibitors in a real world setting study comparing the efficacy of Alirocumab and Evolucumab in patients with heterozygous familial hypercholesterolemia, and both drugs were effective in reducing LDL-c [39].

The cost of PCSK9 inhibitors is the main barrier to treatment. Therefore, these drugs should particularly be used in patients refractory to conventional lipid-lowering therapy [40-47]. A recent Spanish multicenter study was one of the first to demonstrate the cost-effectiveness of using PCSK9 inhibitors in clinical practice [48]. There is a lack of studies in lower-income populations on the cost-effectiveness of such drugs.

Another possible limitation that should be considered when using Evolucumab and Alirocumab to reduce cardiovascular events in clinical practice is adverse effects. Discontinuation due to adverse effects occurred in the included studies for 1.6% to 2.4% of patients who used Evolucumab and 3.6% to 7.2% for Alirocumab [1-3, 9]. In addition, for Evolucumab, there was no statistical difference in relation to placebo for serious adverse effects, neurocognitive events, muscle-related events and hemorrhagic stroke. Only a higher incidence of injection-site reactions was noted [3, 11]. Regarding Alirocumab, adverse events were similar to the placebo group, except for injection-site reactions (3.8% *vs.* 2.1%) in the study by Schwartz *et al*. and myalgia (5.4% *vs.* 2.9%) in the trial by Robinson, *et al*. A meta-analysis of 47 studies that included both Alirocumab and Evolocumab suggests that both drugs are safe and well tolerated, with no increased risk of neurocognitive and neurological adverse events (95% CI 0.913-1.163) [49].

There were few limitations in the review process used, since we included studies with patients of any age, there were no limitations regarding the publication date of the studies, all dosages of both drugs were included, as well as all administration frequencies. No bias was found in the studies using the funnel plots and the analysis carried out using the RoB 2 tool [12] and only randomized clinical trials were included. We do not believe that the dose variation affected our results, as only 1 study with alirocumab used a different dose (150 mg). Furthermore, the baseline characteristics of the studies (age, sex, BMI, are similar. However, there is a significant difference in the follow-up of the studies, something that constitutes a limitation of this systematic review since studies that evaluate cardiovascular outcomes are extremely dependent on the treatment time. The longer the time, the more events will occur.

The results presented have great clinical relevance since cardiovascular events are the main cause of death in the world, and we present 2 drugs with high effectiveness against these outcomes. However, more clinical trials on the topic are necessary in order to produce more high-level scientific research on this topic.

## CONCLUSION

This systematic review was able to compare the effectiveness of 2 PCSK9 inhibitors in reducing cardiovascular outcomes. Alirocumab reduced the occurrence of AMI by 15%, stroke by 25% and hospitalization for unstable angina by 42%, while evolocumab reduced the occurrence of AMI by 25% and coronary revascularization by 19%. However, there was only a statistically significant difference between the drugs in reducing hospitalization for unstable angina, favoring alirocumab. These findings can better guide physicians' decisions about which drug to use. Alirocumab may be a better choice if the patient is at high risk for stroke or MI/hospitalization for unstable angina, while evolocumab may be indicated if one wishes to avoid coronary revascularization. More robust clinical trials are needed to evaluate non-cardiovascular outcomes, such as neuroprotection.

Therefore, it is possible to state that both drugs are effective in reducing some cardiovascular outcomes, with alirocumab being superior to evolocumab, as it was effective in reducing 3 cardiovascular outcomes compared to 2 for evolocumab, in addition to being statistically superior to evolocumab in one of these outcomes.

## Figures and Tables

**Table 1:** General characteristics and cardiovascular outcomes.

**Study**	**Sample Size**	**Drug**	**Dose**	**Myocardial Infarction**	**Coronary** **Revascularization**	**HF**	**Unstable Angina**	**Stroke**	**Death CV Cause**	**Death Any Cause**
Schwartz *et al.*, 2018 [1]	9462	Alirocumab	75 mg	626	731	176	60	111	240	334
	9462	Placebo		722	828	179	37	152	271	392
Robinson *et al.*, 2015 [2]	1553	Alirocumab	150 mg	14	48	9	0	9	4	4
	788	Placebo		18	24	3	1	2	7	7
Schwartz *et al.*, 2023 [10]	730	Alirocumab	75 mg	31	57	-	2	7	11	15
	1460	Placebo		81	89	-	10	21	25	48
Sabatine *et al.*, 2015 [9]	2976	Evolocumab	140 mg or 420 mg	9	15	1	3	3	4	0
	1489	Standart treatment		3	17	1	3	2	3	3
Sabatine *et al.*, 2017 [3]	13,784	Evolocumab	140 mg or 420 mg	468	759	151	236	207	251	444
	13,780	Placebo		639	965	168	239	262	240	426
O’Donoghue *et al.*, 2022 [11]	3355	Evolocumab	140 mg or 420 mg	151	280	-	-	102	107	338
	3280	Placebo		194	313	-	-	94	138	344

## Data Availability

The authors confirm that the data supporting the findings of this study are available within the article.

## LIST OF ABBREVIATIONS

AMI: Acute Myocardial Infarction
BMI: Body Mass Index
HF: Heart Failure
MI: Myocardial Infarction
NAFLD: Non-alcoholic Fatty Liver Disease
RR: Relative Risk

## STANDARDS OF REPORTING

PRISMA guidelines and methodology were followed.

## SUPPLEMENTARY MATERIAL

PRISMA checklist and supplementary material are available on the publisher’s website along with the published article.
